# Effects of SR141716A on Cognitive and Depression-Related Behavior in an Animal Model of Premotor Parkinson's Disease

**DOI:** 10.4061/2010/238491

**Published:** 2010-09-26

**Authors:** M. T. Tadaiesky, P. A. Dombrowski, C. Da Cunha, R. N. Takahashi

**Affiliations:** ^1^Departamento de Farmacologia, Centro de Ciências Biológicas, Universidade Federal de Santa Catarina (UFSC), Campus Universitário, Trindade, Bloco D/CCB, P.O. Box 476, 88040-970 Florianópolis, SC, Brazil; ^2^Laboratório de Fisiologia e Farmacologia do Sistema Nervoso Central, Setor de Ciências Biológicas, Universidade Federal do Paraná (UFPR), Centro Politécnico, 81531-980 Curitiba, PR, Brazil

## Abstract

A previous study from our laboratory revealed that moderate nigral dopaminergic degeneration caused emotional and cognitive deficits in rats, paralleling early signs of Parkinson's disease. Recent evidence suggests that the blockade of cannabinoid CB_1_ receptors might be beneficial to alleviate motor inhibition typical of Parkinson's disease. Here, we investigated whether antagonism of CB_1_ receptors would improve emotional and cognitive deficits in a rat model of premotor Parkinson's disease. Depression-like behavior and cognition were assessed with the forced swim test and the social recognition test, respectively. Confirming our previous study, rats injected with 6-hydroxydopamine in striatum presented emotional and cognitive alterations which were improved by acute injection of SR141716A. HPLC analysis of monoamine levels demonstrated alterations in the striatum and prefrontal cortex after SR141716A injection. These findings suggest a role for CB_1_ receptors in the early symptoms caused by degeneration of dopaminergic neurons in the striatum, as observed in Parkinson's disease.

## 1. Introduction

A growing body of evidence suggests that cognitive and emotional symptoms can precede the classical motor symptoms of Parkinson's disease (PD). Epidemiological, pathological, and clinical studies have provided data in favor of the existence of this premotor phase of PD [1]. Premotor symptoms in PD include mild emotional and cognitive dysfunctions. During this phase, the neuropathological injuries progress without concomitant motor impairments [2]. When cardinal motor signs (bradykinesia, rest tremor, and rigidity) required for PD diagnosis appear, about 60% of dopaminergic neurons in the substantia nigra have been lost, and striatal dopamine content has been reduced by 60%–80% [3]. Recently, we have developed a premotor model of PD in rats, in which emotional and cognitive deficits occur in the absence of motor alterations [4]. In particular, we have shown that the bilateral intrastriatal injection of 6-hydroxydopamine (6-OHDA) in adult rats increases immobility time in the forced swim test one week after drug administration, whereas discrimination impairments in the social recognition test appear three weeks after 6-OHDA injection. 

Endocannabinoids—that is, anandamide and 2-arachidonoylglycerol—are synthesized on demand through cleavage of membrane phospholipids and act as retrograde messengers at central synapses, regulating a number of physiological functions, including emotionality and cognition [5, 6]. These molecules bind to the CB_1_ cannabinoid receptor on presynaptic axon terminals to regulate ion channel activity and inhibit neurotransmitter release [7]. The evidence that the endocannabinoid transmission is overactive in the basal ganglia of humans affected by PD [8] and in rat models of PD [9, 10] suggests that the blockade of cannabinoid CB_1_ receptors might be beneficial to alleviate PD symptoms. In fact, evidence from nonhuman primates and rodents has shown that the administration of SR141716A improves motor symptoms in models of PD [11–14]. These data suggest that CB_1_ receptor-mediated transmission plays a functional role in motor alterations developed in the course of the disease. However, to the best of our knowledge, the effects of CB_1_ receptor antagonism in the early phase of this disorder have not yet been studied. 

The CB_1_ receptors are densely expressed in brain areas controlling emotional and cognitive processes, such as the limbic system and the prefrontal cortex [15]. Therefore, it is possible that the endocannabinoid system mediates the emotional and cognitive alterations in this degenerative disease. For this reason, the present experiments investigated whether the cannabinoid system influences emotional and cognitive alterations in a model of early PD and whether the CB_1_ antagonist SR141716A reduces the 6-OHDA damaging effects in depression-like behavior and memory function in rats.

## 2. Methods

### 2.1. Animals

A total of forty adult (3 months-old) male Wistar rats were used in the forced swim test, while thirty-two adult and six juvenile (1 month-old) male Wistar rats were used in the social recognition test. The animals were housed in a room with controlled photoperiod (07:00–19:00 lights on) and temperature (23 ±1°C). They had free access to standard food and tap water. All procedures used in the present study complied with the guidelines on animal care of the UFSC Ethics Committee on the Use of Animals, which follows the “Principles of laboratory animal care” from NIH.

### 2.2. Intrastriatal Injection of 6-OHDA

The procedure used to induce moderate dopaminergic degeneration in rats was based on the model previously described by us [4] using intrastriatal 6-OHDA injection. All surgical procedures were conducted with aseptic technique. 6-OHDA (12 *μ*g per injection, diluted in 0.9% NaCl, supplemented with 0.1% ascorbic acid, injection volume 2.5 *μ*l at the rate of 0.5 *μ*l/min; Sigma, USA) was injected over 5 minutes bilaterally into the ventrolateral area of the dorsal striatum. Stereotaxic infusion followed the coordinates of the Paxinos and Watson [16] atlas: AP: 1.1 mm, ML: ±3.2 mm, and DV: −7.2 mm from bregma and dura, using a Hamilton 10 *μ*l syringe with a 26-gauge needle connected to a 30-gauge cannula. Following injection, the cannula was left in place for 2 minutes before being retracted to allow complete diffusion of the drug. All animals were treated with i.p. injection of 20 mg/kg desipramine (diluted in 0.9% NaCl, Sigma, USA) 30 minutes before surgery, in order to protect noradrenergic terminals from 6-OHDA toxicity. The stereotaxic surgery was performed under ketamine (15 mg/kg, Dopalen; Agribrands)/xylazine (2.5 mg/kg, Rompun; Bayer) anesthesia. Sham-operated rats were submitted to the same protocol except that vehicle was injected instead of 6-OHDA. Our previous study [4] showed that emotional and cognitive alterations were time dependent in this rat model of PD. Therefore, the behavioral experiments were carried out one (forced swim test) and three (social recognition test) weeks after surgery. Subgroups of 4–8 animals were killed by decapitation immediately following behavioral tests for neurochemical analysis.

### 2.3. Forced Swim Test

The procedure was previously described by Porsolt et al. [17] and established as a standard procedure in our laboratory [4]. Rats were divided into five equal groups (*n* = 8). Beginning about a week after surgery, the rats were acclimatized to the experimental room for at least 30 minutes before test. Rats were placed in individual cylinders (40 cm in height and 17 cm in diameter) containing water (water depth was 30 cm; 25 ±1°C). Two swimming sessions were conducted (an initial 15-min pretest followed by a 5-min test 24 hours later). SR141716A (0.5, 1, and 3 mg/kg, dissolved in saline with 10% DMSO plus 0.1% Tween 80, Sanofi-Aventis, France) and vehicle were administered by i.p. route 30 minutes before the second session of forced swimming. The total immobility time was manually scored for 5 minutes. Immobility was defined as motionless flotation, except for those movements necessary to keep the rat's head above the water.

### 2.4. Social Recognition Test

Short-term social memory was assessed with the social recognition task as previously established in our laboratory [4, 18]. Around three weeks following surgery, 6-OHDA-injected and sham rats were housed individually in standard plastic cages (42 × 34 × 17 cm) and were used after three days of habituation to their new environment. Each animal was injected with the CB_1_ antagonist (0.5 and 1 mg/kg) or vehicle 30 minutes before the social recognition memory test (*n* = 8). The test was scored in an observation room, to which the rats had been habituated for 30 minutes before the test began. All juveniles were isolated in individual cages for 30 minutes prior to the beginning of the experiment. The social recognition test consisted of two successive 5-minute presentations separated by a 30-minute interval, where a juvenile rat was placed in the home cage of the isolated adult rat. Time spent investigating the juvenile (nosing, sniffing, grooming, or pawing) was recorded during each session. At the end of the first presentation, the juvenile rat was removed and kept in an individual cage during the delay period and re-exposed to the adult rat after 30 minutes. 

### 2.5. Neurochemical Study

For determining DA, 3,4-dihydroxyphenylacetic acid (DOPAC), homovallinic acid (HVA), NA, and 5-HT contents in brain, the rats were killed by decapitation one and three weeks after 6-OHDA administration.

The rats were decapitated, brains were immediately removed, and striatum, prefrontal cortex, and hippocampus were freshly dissected, frozen in liquid nitrogen, and stored at −70°C. The concentrations of DA, DOPAC, HVA, NA, and 5-HT in striatum, prefrontal cortex, and hippocampus were assayed by reverse-phase HPLC with electrochemical detection (ED). The system consisted of a Synergi Fusion-RP C-18 reverse-phase column (150 × 4.6 mm i.d., 4 *μ*m particle size) fitted with a 4 × 3.0 mm precolumn (SecurityGuard Cartridges Fusion-RP), an electrochemical detector (ESA Coulochem III Electrochemical Detector) equipped with a guard cell (ESA 5020), with the electrode set at 350 mV and a dual electrode analytical cell (ESA 5011A); and a LC-20AT pump (Shimadzu) equipped with a manual Rheodyne 7725 injector with a 20 *μ*l loop. The column was maintained inside a temperature-controlled oven (25°C, Shimadzu). The cell had two chambers in series: each chamber included a porous graphite colorimetric electrode, a double counter electrode, and a double reference electrode. Oxidizing potentials were set at 100 mV for the first electrode and at 450 mV for the second electrode. DA, DOPAC, HVA, NA, and 5-HT were detected at the second electrode. The tissue samples were homogenized with an ultrasonic cell disrupter (Sonics) in 0.1 M perchloric acid containing sodium metabisulfite 0.02% and internal standard. After centrifugation at 10000 × g for 30 minutes at 4°C, 20 *μ*l of the supernatant was injected into the chromatograph. The mobile phase used at a flow rate of 1 ml/min had the following composition: 20 g citric acid monohydrate (Merck), 200 mg octane-1-sulfonic acid sodium salt (Merck), 40 mg ethylenediaminetetraacetic acid (EDTA) (Sigma), and 900 ml HPLC-grade water. The pH of the buffer running solution was adjusted to 4.0 then filtered through a 0.45 *μ*m filter. Methanol (Merck) was added to give a final composition of 10% methanol (v/v). The peak areas of the external standards were used to quantify the sample peaks.

### 2.6. Statistics

Statistically significant differences in the forced swim test were assessed by one-way analysis of variance (ANOVA), followed by Newman-Keuls post hoc test. Two-way repeated-measure ANOVA was used in the social recognition test, followed by Bonferroni's test for multiple comparisons. Statistical analysis of HPLC measures was carried out by unpaired Student's *t*-test. All values were expressed as mean ± SEM. Statistical significance was defined as *P *≤ .05.

## 3. Results

**Figure 1:**
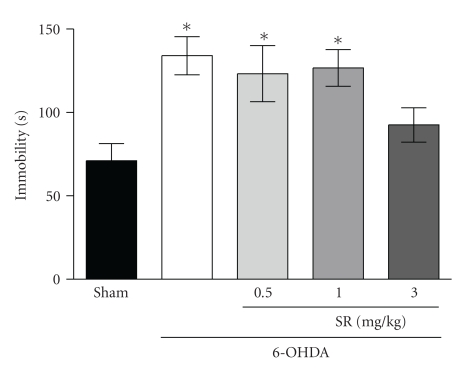
Effects of acute administration of SR141716A (0.5, 1, and 3 mg/kg), i.p. 30 minutes before the second session of forced swim test on 6-OHDA-treated rats. Results are expressed as mean immobility time ± SEM in comparison to sham controls. Statistical analysis was performed by one-way ANOVA followed by Newman-Keuls post hoc test. *P ≤ .05 compared to the sham group (*n* = 8 per group).

In the forced swim test (Figure 1), one-way ANOVA revealed an increase in immobility time compared to the sham group (*F*_4,50_ = 4.01; *P *< .05). Acute treatment with SR141716A (3 mg/kg, i.p.) reverted the increase in immobility time induced by the 6-OHDA treatment. 

After the forced swim test, the neurotransmitter levels of the brain striatum, prefrontal cortex, and hippocampus were measured in the 6-OHDA-treated rats and the 6-OHDA-treated rats that received the effective dose of SR141716A (3 mg/kg). The measures of brain monoamine levels showed a significant increase in the levels of DA (*t*_6_ = 2.28; *P *< .05), DOPAC (*t*_6_ = 2.35; *P *< .05), and HVA (*t*_6_ = 2.18; *P *< .05) in the 6-OHDA-lesioned rats treated with SR141716A. No significant difference was found in the striatal levels of NA (*t*_6_ = 1.38; *P *> .05) and 5-HT (*t*_6_ = 1.51; *P *> .05) and in the levels of DA, DOPAC, HVA, NA, and 5-HT in the prefrontal cortex and hippocampus of the 6-OHDA-lesioned rats treated with SR141716A compared to the 6-OHDA-lesioned rats that received vehicle. Data are shown in Table 1.

**Figure 2:**
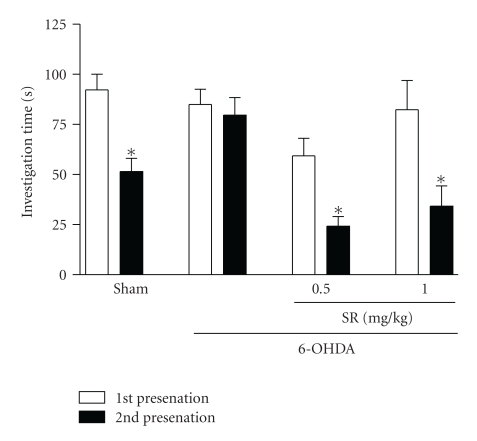
Effects of acute administration of SR141716A (0.5 and 1 mg/kg) i.p. 30 minutes before social recognition test on 6-OHDA-treated rats. Results are expressed as mean investigation time ± SEM in comparison to sham controls. Statistical analysis was performed by two-way repeated-measure ANOVA followed by Bonferroni's post hoc test. *P ≤ .05 compared to the first juvenile presentation (*n* = 8 per group).

In the social recognition test procedure, two-way ANOVA (treatment versus juvenile presentation) revealed no significant effect for interaction factor (*F*_3,32_ = 2.31; *P *= .08). However, it indicated a significant effect for drug treatment (*F*_3,32_ = 27.3; *P *< .0001) and for juvenile presentation, that is, the second presentation of the juvenile rat (*F*_3,32_ = 8.0; *P *< .0001). Bonferroni's post hoc test showed that 6-OHDA-treated rats presented a clear impairment of the juvenile recognition ability in comparison to sham rats, since the 6-OHDA group spent as much time investigating the juvenile rat during the second encounter as they did on the first exposure. 6-OHDA + SR groups spent less time investigating the juvenile rat during the second exposure compared to the first one. Data are shown in Figure 2.

After the social recognition test, the neurotransmitter levels of the brain striatum, prefrontal cortex, and hippocampus were measured in the 6-OHDA-treated rats and the 6-OHDA-treated rats that received the effective dose of SR141716A (0.5 mg/kg). NA (*t*_6_ = 3.09; *P *< .05) and 5-HT (*t*_6_ = 2.44; *P* = .05) levels in the prefrontal cortex were significantly increased in the SR-treated group. There were no significant differences between the two groups in the striatum and hippocampus (Table 2).

## 4. Discussion

Rats with bilateral 6-OHDA lesions of the nigrostriatal pathway displayed depressive-like behavior and social memory impairment, modeling the pathophysiology of nonmotor alterations that occur early in PD patients [1]. The evidence of an increase in the endocannabinoid transmission in the basal ganglia in patients and animal models of this disease [8, 10] supports the potential of SR141716A or other CB_1_ receptor antagonists to alleviate PD symptoms. The data found in this paper confirm this hypothesis, since we showed that blockade of CB_1_ receptors significantly attenuated 6-OHDA-induced impairments in the forced swim and social recognition tests. This was accompanied by an increase in DA levels in the striatum and NA and 5-HT levels in the prefrontal cortex.

In the forced swim test, 6-OHDA-treated rats displayed a greater tendency toward despair behavior compared to sham animals and those treated with SR141716A, therefore suggesting that the blockade of CB_1_ receptors restores the normal coping response when animals are exposed to inescapable aversive stimuli. The reduction of 6-OHDA-induced increase in immobility in the forced swim test by SR141716A was evident at the dose of 3 mg/kg but not at lower doses (0.5 and 1 mg/kg), suggesting that low doses of this CB_1_ antagonist may not be sufficient to induce antidepressant-like effect in this animal model of PD. It must be conceded, however, that rimonabant can induce hypermotility in rodents at high doses, and this could have affected the outcome of our results. Nevertheless, the present evidence agrees with the antidepressant-like activity reported by Griebel et al. [19], in which SR141716A was shown to reduce immobility time in the forced swim test at the dose of 3 mg/kg, but not at 1 mg/kg in rats. 

In fact, although this, to our knowledge, is the first report on the behavioral action of a CB_1_ receptor antagonist in a model of PD-associated depressive symptoms, the profile of CB_1_ antagonists on emotionality has been demonstrated in other rodent models of depression. Antidepressant-like effects have been established in models using mice, rats, and gerbils, and in models using different dependent measures [19–21]. Confirmatory evidence of the involvement of CB_1_ receptors in the antidepressant-like effects of SR141716A and another CB_1_ antagonist, AM251, comes from their receptor specificity for CB_1_ receptors and from pharmacological agonist interaction studies. Thus, the direct-acting CB_1_ receptor agonist CP55940 prevented the antidepressant-like effects of AM251. Another crucial piece of data tying CB_1_ receptors to the antidepressant-like mechanism of action of these compounds comes from the demonstration that CB_1_ receptor-null mice do not display the antidepressant-like effects of AM251 [20], demonstrating the importance of this receptor to the antidepressant-like effect of these cannabinoid antagonists.

Our results suggest that the mechanisms underlying the antidepressant-like effects of SR141716A possibly involve restoration of dopamine levels in the striatum, since acute administration of 3 mg/kg of SR141716A produced elevations in DA, DOPAC, and HVA levels in the striatum of 6-OHDA-treated rats. In line with this are the findings from our previous study showing that the increase in immobility time in the forced swim test after 6-OHDA treatment was accompanied by a reduction in the levels of DA and its metabolites in the striatum [4]. The increase in the striatal levels of DA was most probably due to the blockade of CB_1_ receptors localized on striatonigral GABAergic neurons rather than to a direct effect of this cannabinoid antagonist on nigrostriatal dopaminergic projections. In fact, nigrostriatal dopaminergic neurons do not contain CB_1_ receptors, at least in the adult brain, although these receptors are co-localized with D1 and D2 dopaminergic receptors in striatal projections [22]. 

Interestingly, the 0.5 mg/kg SR141716A administration led to a significant increase in NA and 5-HT levels in the prefrontal cortex, but caused no alterations in striatal neurotransmitters. At the dose of 3 mg/kg, however, DA and its metabolites levels were significantly increased in striatum, while no alterations were seen in prefrontal cortex neurotransmitter levels. This divergent increase in neurotransmitter levels in different brain structures seems to be caused by the doses used in this study and deserves to be better investigated, perhaps employing other doses of this CB_1_ antagonist in both 6-OHDA and sham animals.

Despite the strong evidence indicating a beneficial effect of CB_1_ receptor antagonists in depression, the data obtained so far do not completely support this assumption since, while we and others [19–21] have demonstrated that SR141716A might be effective to reduce depressive-like symptoms in animal models, this CB_1_ antagonist was withdrawn from the market as an antiobesity drug because of its prodepressant effects. On the other side, in a clinical trial with SR141716A in schizophrenic patients [23], in which the drug did not affect the efficacy of endpoints, no adverse effects on mood and anxiety were reported. Clinical investigation specifically designed to address the antidepressant potential of this mechanism is, therefore, the only resolution to the question of efficacy.

The acute SR141716A effect on short-term memory functioning in this model of premotor PD was assessed using the social recognition test. Social recognition memory bases on olfactory cues and represents a form of olfactory short-term memory. In this task, if the delay period is less than 40 minutes, the adult male rat usually displays recognition of the juvenile rat by significantly reducing the social investigation time during the second presentation compared to the first one. The ability of social discrimination was disrupted in rats treated with 6-OHDA, an effect reversed by SR141716A. 

It is consistent that CB_1_ cannabinoid receptors are involved in cognitive processes including memory in both human and animals [24–26]. A study from Micale et al. [27] showed that SR141716A counteracted amnesic effects in beta amyloid-injected mice, an Alzheimer's disease model, strengthening the proposal role of SR141716A on the treatment of neurodegenerative diseases.

 Terranova et al. [28] previously demonstrated that SR141716A facilitates short-term memory in the social recognition test. Here, we extend these data by showing that SR141716A is able to improve short-term recognition memory in 6-OHDA-lesioned animals. It can therefore be hypothesized that this drug may be useful to treat memory deficits associated with PD. In our previous study [4], social recognition deficits were accompanied by alterations in neurotransmitter levels in the prefrontal cortex. Here, those results further imply this area in this kind of memory, since improvement in recognition by SR141716A was associated with increases in the levels of NA and 5-HT in the prefrontal cortex.

## 5. Conclusions

In conclusion, the preponderance of data presented here suggests some relationship between the acute effects of SR141716A on the forced swim and social recognition tests and a therapeutic potential in the treatment of behavioral alterations in PD, further strengthening the therapeutic value of this CB_1_ receptor antagonist which was already proposed for the treatment of motor impairments in PD [12, 29].

## Figures and Tables

**Table 1:** Effects of acute administration of SR141716A (3 mg/kg) i.p. on neurotransmitter levels in the striatum, prefrontal cortex, and hippocampus one week after 6-OHDA administration. Results are expressed as mean ± SEM in comparison to the 6-OHDA group. Statistical analysis was performed by unpaired Student's *t*-test.

Brain region	Group	Content (ng/mg protein)				
		DA	DOPAC	HVA	NA	5-HT
Striatum	6-OHDA	1685 ± 253.6	932.1 ± 101.7	600.8 ± 111.1	119.5 ± 7.5	228.5 ± 15.4
	6-OHDA + SR	2869 ± 451.7*	1491 ± 214*	1043 ± 169.6*	135.1 ± 8.4	275.3 ± 26.5
Prefrontal cortex	6-OHDA	17.7 ± 6.8	20 ± 8.9	23.02 ± 8.7	144.2 ± 29.5	266.9 ± 68.1
	6-OHDA + SR	8.7 ± 1.9	23.7 ± 7.9	24.24 ± 11.2	155.9 ± 7.9	387.6 ± 95.5
Hippocampus	6-OHDA	30.9 ± 1.9	22.2 ± 2.5	516.4 ± 493.7	237.8 ± 34.3	104 ± 34
	6-OHDA + SR	30.4 ± 1.3	18.2 ± 3	45 ± 6.2	273.8 ± 42.2	167.2 ± 50.2

*P ≤ .05 compared to the 6-OHDA group (*n* = 4 per group).

**Table 2:** Effects of acute administration of SR141716A (0.5 mg/kg) i.p. on neurotransmitter levels in the striatum, prefrontal cortex, and hippocampus three weeks after 6-OHDA administration. Results are expressed as mean ± SEM in comparison to the 6-OHDA group. Statistical analysis was performed by unpaired Student's *t*-test.

Brain region	Group	Content (ng/mg protein)				
		DA	DOPAC	HVA	NA	5-HT
Striatum	6-OHDA	1364 ± 337.2	1266 ± 223	777.2 ± 107.8	131 ± 19.5	227.9 ± 15.7
	6-OHDA + SR	2099 ± 759.9	1190 ± 61.8	828.4 ± 74.4	125.7 ± 6.5	248.1 ± 32.2
Prefrontal cortex	6-OHDA	16.8 ± 4.1	20.2 ± 11.7	35.1 ± 7.5	104.9 ± 29	198.4 ± 107.1
	6-OHDA + SR	17.1 ± 4.7	12.3 ± 5.4	42.5 ± 5.3	208.7 ± 16.6*	467.3 ± 24.9*
Hippocampus	6-OHDA	23.4 ± 7.8	17.6 ± 2.8	53.9 ± 14.2	195.9 ± 44.4	116.4 ± 59.6
	6-OHDA + SR	28 ± 4.4	20.3 ± 2.5	32.8 ± 8.5	297.5 ± 28.2	165.6 ± 41.6

*P ≤ .05 compared to the 6-OHDA group (*n* = 4 per group).

## References

[1] Tolosa E, Poewe W (2009). Premotor Parkinson disease. *Neurology*.

[2] Becker G, Müller A, Braune S (2002). Early diagnosis of Parkinson’s disease. *Journal of Neurology, Supplement*.

[3] Tissingh G, Bergmans P, Booij J (1998). Drug-naive patients with Parkinson’s disease in Hoehn and Yahr stages I and II show a bilateral decrease in striatal dopamine transporters as revealed by [123I]*β*-CIT SPECT. *Journal of Neurology*.

[4] Tadaiesky MT, Dombrowski PA, Figueiredo CP, Cargnin-Ferreira E, Da Cunha C, Takahashi RN (2008). Emotional, cognitive and neurochemical alterations in a premotor stage model of Parkinson’s disease. *Neuroscience*.

[5] Lutz B (2009). Endocannabinoid signals in the control of emotion. *Current Opinion in Pharmacology*.

[6] Wotjak CT (2005). Role of endogenous cannabinoids in cognition and emotionality. *Mini-Reviews in Medicinal Chemistry*.

[7] Piomelli D (2003). The molecular logic of endocannabinoid signalling. *Nature Reviews Neuroscience*.

[8] Pisani A, Fezza F, Galati S (2005). High endogenous cannabinoid levels in the cerebrospinal fluid of untreated Parkinson’s disease patients. *Annals of Neurology*.

[9] Di Marzo V, Hill MP, Bisogno T, Crossman AR, Brotchie JM (2000). Enhanced levels of endogenous cannabinoids in the globus pallidus are associated with a reduction in movement in an animal model of Parkinson’s disease. *FASEB Journal*.

[10] Gubellini P, Picconi B, Bari M (2002). Experimental parkinsonism alters endocannabinoid degradation: implications for striatal glutamatergic transmission. *Journal of Neuroscience*.

[11] Fernandez-Espejo E, Caraballo I, de Fonseca FR (2005). Cannabinoid CB1 antagonists possess antiparkinsonian efficacy only in rats with very severe nigral lesion in experimental parkinsonism. *Neurobiology of Disease*.

[12] González S, Scorticati C, García-Arencibia M, de Miguel R, Ramos JA, Fernández-Ruiz J (2006). Effects of rimonabant, a selective cannabinoid CB1 receptor antagonist, in a rat model of Parkinson’s disease. *Brain Research*.

[13] van der Stelt M, Fox SH, Hill M (2005). A role for endocannabinoids in the generation of parkinsonism and levodopa-induced dyskinesia in MPTP-lesioned non-human primate models of Parkinson’s disease. *FASEB Journal*.

[14] Cao X, Liang L, Hadcock JR (2007). Blockade of cannabinoid type 1 receptors augments the antiparkinsonian action of levodopa without affecting dyskinesias in 1-methyl-4-phenyl-1,2,3,6- tetrahydropyridine-treated rhesus monkeys. *Journal of Pharmacology and Experimental Therapeutics*.

[15] Mackie K (2005). Distribution of cannabinoid receptors in the central and peripheral nervous system. *Handbook of Experimental Pharmacology*.

[16] Paxinos G, Watson C (2005). *The Rat Brain in Stereotaxic Coordinates*.

[17] Porsolt RD, Anton G, Blavet N, Jalfre M (1978). Behavioural despair in rats: a new model sensitive to antidepressant treatments. *European Journal of Pharmacology*.

[18] Prediger RDS, Takahashi RN (2003). Ethanol improves short-term social memory in rats. Involvement of opioid and muscarinic receptors. *European Journal of Pharmacology*.

[19] Griebel G, Stemmelin J, Scatton B (2005). Effects of the cannabinoid CB1 receptor antagonist rimonabant in models of emotional reactivity in rodents. *Biological Psychiatry*.

[20] Shearman LP, Rosko KM, Fleischer R (2003). Antidepressant-like and anorectic effects of the cannabinoid CB1 receptor inverse agonist AM251 in mice. *Behavioural Pharmacology*.

[21] Tzavara ET, Davis RJ, Perry KW (2003). The CB1 receptor antagonist SR141716A selectively increases monoaminergic neurotransmission in the medial prefrontal cortex: implications for therapeutic actions. *British Journal of Pharmacology*.

[22] Herkenham M, Lynn AB, de Costa BR, Richfield EK (1991). Neuronal localization of cannabinoid receptors in the basal ganglia of the rat. *Brain Research*.

[23] Meltzer HY, Arvanitis L, Bauer D, Rein W (2004). Placebo-controlled evaluation of four novel compounds for the treatment of schizophrenia and schizoaffective disorder. *American Journal of Psychiatry*.

[24] Hampson RE, Deadwyler SA (1999). Cannabinoids, hippocampal function and memory. *Life Sciences*.

[25] Lutz B (2007). The endocannabinoid system and extinction learning. *Molecular Neurobiology*.

[26] Deadwyler SA, Goonawardena AV, Hampson RE (2007). Short-term memory is modulated by the spontaneous release of endocannabinoids: evidence from hippocampal population codes. *Behavioural Pharmacology*.

[27] Micale V, Cristino L, Tamburella A (2010). Enhanced cognitive performance of dopamine D3 receptor " knock-out" mice in the step-through passive-avoidance test: assessing the role of the endocannabinoid/endovanilloid systems. *Pharmacological Research*.

[28] Terranova J-P, Storme J-J, Lafon N (1996). Improvement of memory in rodents by the selective GB1 cannabinoid receptor antagonist, SR 141716. *Psychopharmacology*.

[29] Brotchie JM (2003). CB1 cannabinoid receptor signalling in Parkinson’s disease. *Current Opinion in Pharmacology*.

